# The Impact of Different Degrees of Feedback on Physical Activity Levels: A 4-Week Intervention Study

**DOI:** 10.3390/ijerph120606561

**Published:** 2015-06-09

**Authors:** Karen Van Hoye, Filip Boen, Johan Lefevre

**Affiliations:** Department of Kinesiology, Physical Activity, Sports & Health Research Group, KU Leuven, Leuven 3001, Belgium; E-Mails: Filip.Boen@faber.kuleuven.be (F.B.); Johan.Lefevre@faber.kuleuven.be (J.L.)

**Keywords:** physical activity assessment, motion sensors, exercise psychology, health promotion

## Abstract

Assessing levels of physical activity (PA) and providing feedback about these levels might have an effect on participant’s PA behavior. This study discusses the effect of different levels of feedback—from minimal to use of a feedback display and coach—on PA over a 4-week intervention period. PA was measured at baseline, during and immediately after the intervention. Participants (*n* = 227) were randomly assigned to a Minimal Intervention Group (MIG-no feedback), Pedometer Group (PG-feedback on steps taken), Display Group (DG-feedback on steps, minutes of moderate to vigorous physical activity and energy expenditure) or Coaching Group (CoachG-same as DG with need-supportive coaching). Two-way ANCOVA showed no significant Group × Time interaction effect for the different PA variables between the MIG and PG. Also no differences emerged between PG and DG. As hypothesized, CoachG had higher PA values throughout the intervention compared with DG. Self-monitoring using a pedometer resulted in more steps compared with a no-feedback condition at the start of the intervention. However, adding individualized coaching seems necessary to increase the PA level until the end of the intervention.

## 1. Introduction

The goal of physical activity (PA) interventions is to help participants change their behavior (*i.e.*, increase their PA participation), for example, by modifying their beliefs, attitudes or knowledge of the behavior [1]. The largest health benefits have been observed in insufficiently active people who start doing regular moderate exercise [1]. Therefore, public health policies should focus on encouraging those who are insufficiently active to become moderately active. Those who do not engage in regular PA should begin by incorporating PA into their day, building up gradually to 30 min of moderate intensity activity per day. For insufficiently active people, moderate intensity PA might be easier to begin with and will be more likely to be continued regularly than vigorous PA [2,3,4,5]. Therefore, health experts are broadening their conceptualization of PA from leisure-time activity to ‘a lifestyle or way of life that integrates PA into the daily life routine’ [6].

Because regular PA is a repetitive and complex behavior [7] that involves dynamic interactions among intrapersonal, interpersonal and environmental factors, individuals need to adopt self-regulatory strategies to integrate PA into daily life routine and overcome barriers to increase adherence [8]. Therefore, when individuals start to exercise, they not only need to learn physical skills to help them perform exercise correctly, but also behavioral skills to adhere to the exercise behavior [9].

The behavioral change process can be facilitated by various techniques, which can be categorized along a continuum ranging from passive information to initiatives that more actively seek to support behavioral change [10]. Because the evidence suggest that information provision alone is unlikely to be sufficient to motivate sustainable behavior change, a more proactive technique such as self-monitoring is recommended [11,12]. Self-monitoring implies that individuals are aware of their current PA behaviors and are able to track their performance in relation to the prevailing PA recommendations [13].

In their refined taxonomy of behavior change techniques for PA, Michie *et al.* [14] made a distinction between self-monitoring of behavior (e.g., daily step counts) and self-monitoring of behavioral outcome (e.g., daily energy expenditure). Objective measures such as the pedometer provide immediate feedback on the walking behavior [15], which gives the individual information on attaining a particular step goal (e.g., 10,000 steps per day [16]). A review of literature has already shown that using pedometers as a self-monitoring tool increases PA by approximately 2000 [17] to 2500 [18] steps per day. Baker *et al.* [19] examined the use of pedometers to increase participants’ PA behavior during a short-term 4-week walking intervention. Participants who wore a pedometer increased their step count from baseline to week four (3006 steps/day, *p* < 0.001). Their study highlighted that a personalized goal-setting program and using baseline values was sufficient to produce short-term increases in walking [20].

When the total volume of activity or the energy expenditure (EE) are the desired outcome variables, more sophisticated accelerometer-based devices are needed [21]. The SenseWear Armband (SWA, BodyMedia, Inc. Pittsburgh, PA, USA) is a PA monitor that is worn on the upper arm and that receives information from different sensors to estimate EE [22]. SenseWear^®^ Professional facilitates the evaluation of the test person’s life style, physical activity, rest and sleep patterns, allowing new insights based on evidence rather than assessment by cumbersome and error prone diaries or logs. The graphical display of information can have a fundamental role in helping to foster understanding the physical activity behavior [23].

The Display is an optional accessory intended for use with the Armband. It allows users to easily view up-to-the-minute information including total EE, steps taken, and physical activity duration. Consequently, when using the SWA display, daily targets can be set both on the behavior (e.g., number of steps and minutes of moderate to vigorous PA (MVPA)) as well as on the behavioral outcome (e.g., total EE per day). When one achieve one of the daily targets, the Display will notify the user with a series of beeps and a scrolling message stating which target has been reached. To our knowledge, no study has so far compared the effectiveness of receiving feedback on the behavior *versus* receiving feedback on the behavioral outcome in the PA domain. Furthermore, no study has ever investigated the added value of receiving messages when targets are met.

There are several theories within exercise psychology that are closely related to the self-monitoring of PA. A motivational theory that has received a lot of research attention over the past few years in predicting PA as well as in the development of PA interventions is the Self-Determination Theory (SDT) [24]. SDT research has focused on the importance of need-supportive coaching in facilitating autonomous motivation (e.g., engaging in an activity with eagerness and volition, with a sense of choice and willingness) for leisure time PA. Need-supportive coaches provide individuals with feedback about their activity behavior and in that way satisfy the individual’s need for autonomy, competence and relatedness [25]. Depending on how individuals interpret information obtained from the coaches’ feedback, they can become autonomously motivated, which consequently enhances the likelihood of PA enjoyment, engagement, and persistence. To our knowledge, no study has yet investigated the added value of need-supportive coaching to a self-monitoring device in changing the PA behavior.

Therefore, this randomized controlled study had four objectives. The first objective was to find support for the effectiveness of feedback on the behavior (e.g., steps) by comparing a pedometer *versus* a no feedback condition. A second objective was to compare the effectiveness of feedback on the behavior only (e.g., steps) *versus* feedback on both the behavior (e.g., steps and minutes of MVPA) and the behavioral outcome (e.g., total EE per day). The third objective was to examine whether a need-supportive climate provided by a Personal Coach would have an additional effect on PA behavior change when it is combined with giving real-time feedback on both the behavior (e.g., steps and minutes of MVPA) and the behavioral outcome (e.g., total EE per day).

PA intervention studies often report outcome measures at baseline and at completion of the intervention. In most of these studies, the weekly change of the activity behavior during the intervention is lacking. Therefore, a fourth and final objective of the present study was to explore the weekly change of the PA behavior when using different degrees of PA feedback in previously inactive working adults.

## 2. Experimental Section

### 2.1. Study Design

A 12-month randomized controlled trial study was conducted consisting of four intervention arms varying in different degrees of feedback. PA data were objectively collected during a baseline measurement, weekly during the 4-week intervention period, one week after the intervention (post) and again at 3, 6 and 12 months after randomization. To maximize motivation and minimalize drop-out, we decided to use a 4-week intervention instead of a longer intervention period, because participants had to wear the SWA all-day long. In this paper, the weekly results of measurement feedback throughout the intervention and the PA behavior one week after the intervention will be discussed. The main variables of interest were mean daily steps, mean minutes of MVPA (minutes of PA above three metabolic equivalents (METs)), mean daily total EE, mean active EE (EE for all activities above three METs), and mean daily PA level. The protocol was approved by the Medical Ethics Committee. Each participant signed an informed consent. The trial is registered at www.clinicaltrial.gov (number NCT01432327).

### 2.2. Participants

**Figure 1:**
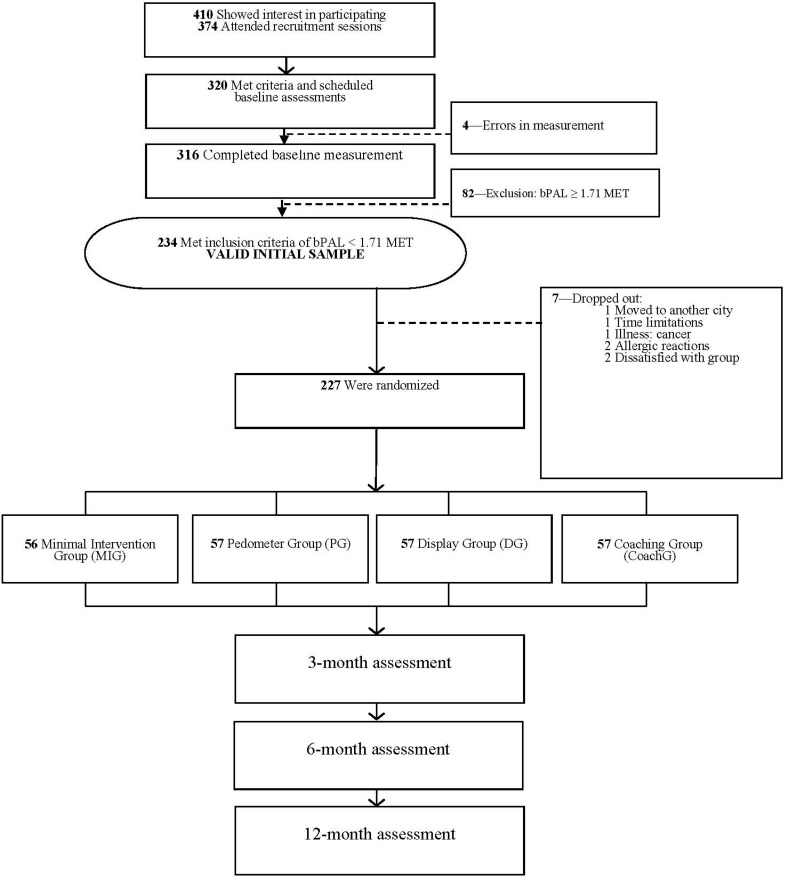
Participants flow from screening to randomization. Abbreviations: bPAL, Physical Activity Level at baseline; MET, Metabolic Equivalent.

Figure 1 shows the flow of participants throughout the trial. Male and female working adults, aged between 19 and 67 years, who mentioned not being physically active during the last year were recruited through flyers, pharmacists, and word of mouth. Full details on participant recruitment and study procedures for the data collection have been described elsewhere [26]. Between July 2010 and July 2011, 410 study participants showed interest in entering the study while 316 individuals completed baseline measurement. Of those 316 participants, 234 individuals met the inclusion criteria of having a baseline PA level of below 1.7 METs, as measured by the SWA and 227 participants were subsequently randomized over the four intervention arms by choosing a card blindfold in a deck of playing cards, where each symbol (clubs, diamonds, hearts and spades) represented an intervention group. The daily PA level (ratio of total EE over resting EE) can be used to classify individuals as either active or inactive. It seems likely that the achievement of 1.7 METs is needed to prevent the transition to overweight or obesity [27], which is one of the first and important consequences of a physically inactive lifestyle. The daily targets of ≥30 min of MVPA (bouts) and 1.7 METs are commonly used and are embedded in two official PA recommendations by the World Health Organization [28,29].

### 2.3. Intervention

#### 2.3.1. Intervention Arms

A random sample of male (*n* = 103) and female (*n* = 124) working adults were randomly assigned to one of four intervention groups: (1) MIG—this group received no feedback during the 4-week intervention period. This group is called a ‘minimal’ intervention group instead of a ‘control’ group because they had a meeting with the test instructor before entering the 4-week intervention period. During this meeting, their objectively measured PA level was discussed and compared with national PA recommendations; (2) PG—this group received information on their daily step count during the 4-week intervention by using a pedometer and was given a step diary to write down their daily steps. They were instructed to take at least 10,000 steps a day; (3) DG—this group received feedback on steps, minutes of MVPA per day and total EE per day during the intervention by means of a real-time wrist watch SWA display and were required to complete a PA diary daily with information on step counts attained, minutes of MVPA, total kcal burned and type of activity performed. They also received a list of possible activities they could perform to increase their energy expenditure; (4) CoachG—this group also received the SWA display and PA diary. The daily targets on steps, minutes of PA and total EE were weekly increased so that at the start of the intervention targets were easily met and by the end of the intervention, more effort was needed. Additionally these individuals had weekly meetings with a Personal Coach to discuss their PA behavior (as written down in their PA diary) and the efforts that were made to change that behavior. Furthermore, the graphical display of information was used to help foster understanding of the PA behavior.

**Figure 2:**
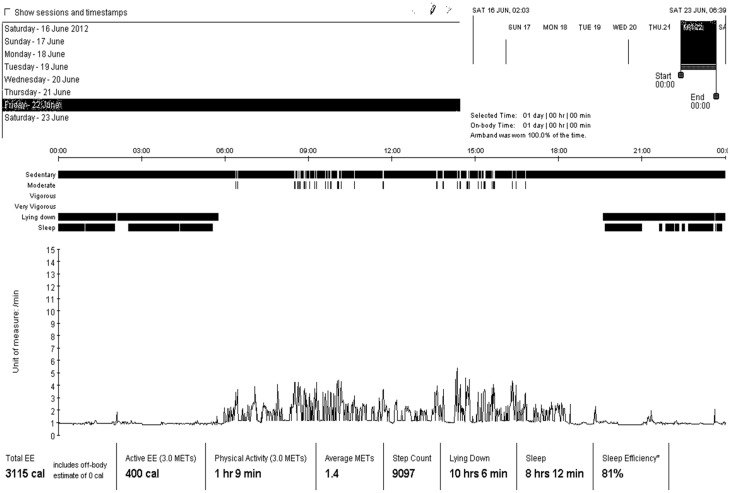
Pattern over 1 day.

Figure 2 shows the output from the SenseWear software and shows a display of selected parameters over a period of one week. Any arbitrary period and any combination of parameters can be selected for display. In this figure, one day (more specifically, Friday the 22th of June) is highlighted. In the on-screen visualizations, the selected parameters are superimposed and displayed on a timeline. Beneath the graphical visualization of the energy expenditure, summary information is given on total EE, active EE, duration of PA, average METs, step count, time lying down, sleep and sleep efficiency over the selected period. Over the 24-hour period, total EE was 3115 calories and 400 calories were spent during activities of at least moderate intensity. The subject spent 1 hour 9 minutes at physical activity (all at moderate exercise level, defined as 3.0–6.0 METS) and had an average activity level of 1.4 METs. Total step count over the 24 hours was 9097 steps. The subject had been lying down for 10 hours 6 minutes and slept for 8 hours 12 minutes which resulted in a sleep efficiency of 81%.

The coach was an academic master in Physical Education and Movement Sciences. Each coaching session lasted between 30 and 45 min and consisted of a check-in of SenseWear data, a discussion of the results, an evaluation of the individual targets, a summary of the current session and a preview of the next session. The present study consisted of only one coach to control for personal bias because each instructor may have their own ways of giving feedback.

#### 2.3.2. Need-Supportive Coaching

The personalized feedback provided as part of the intervention (CoachG) was based on the Self Determination Theory (SDT) of Deci and Ryan [24]. Need-supportive coaching is considered to foster autonomously motivated behavior. During the weekly conversation with the coach, SWA data was exported and the PA behavior was graphically displayed for further discussion. Participants could see their average step count, minutes spent at MVPA and active and total EE. The coach used need-supportive strategies and provided participants with choice (“what kind of activities would you like to do during lunch break?”), opportunities for initiative-taking (“which type of exercises have you done during the past week that were fun?”) and constructive feedback (“you really did a nice job spending more time at PA during the weekend. Maybe now you can try to do this also on a workday? You will see that by doing so you will have more energy managing other tasks during the week”). These strategies were intended to make the participants experience a feeling of autonomy (e.g., that they were the regulator of their own actions), competence (e.g., that they felt capable of attaining the PA goals) and relatedness (e.g., that they experienced care and concern from the coach).

### 2.4. Measures

PA outcome variables were measured by means of the SWA, a multi-sensor activity monitor which has previously been shown to accurately estimate EE during light to moderate intensity activities [30,31]. The monitor was set to record at 1-min epochs. At baseline and one week after the intervention, data collection occurred on seven consecutive days and participants were asked to wear the SWA 24/7 and to remove them while bathing, showering and swimming. To reduce participant dropout and to stimulate adherence, participants were allowed to take off the SWA during sleeping hours during the 4-week intervention period. SWA data cleaning was performed using SAS 9.2 (SAS Institute, Cary, NC, USA). Missing values due to water or sleeping-activities were imputed with the corresponding energy values according to the table of Ainsworth [32]. Each day with more than 5% of missing data (equivalent to 72 min of no data) was excluded from the analysis. PA outcome variables were calculated per week and only included days with at least 95% of data (after imputing missing values for sleeping or water activities). The missing days were consistent between the different intervention arms.

After baseline and after post-intervention assessment, participants PA behavior was discussed in a one-to-one conversation with the test instructor. They received written feedback including information on their objectively measured daily PA level compared with the current PA recommendations by using percentile score forms. During the intervention period, all groups with the exception of the MIG, received real-time feedback about their activity behavior. The PG received feedback on the number of steps by means of a SW digi-walker (Yamax, Tokyo, Japan) a waist-mounted device that is most widely used in research studies. Previous research has identified this pedometer as one of the most accurate and reliable electronic pedometers available [33,34]. The DG and CoachG used the SWA display which provided feedback on both the activity behavior (*i.e.*, daily steps, minutes of MVPA) as well as the behavioral outcome (*i.e.*, daily EE).

### 2.5. Analysis

A pilot study of 73 individuals was conducted to provide preliminary evidence of the efficacy of PA feedback on the PA behavior. G × power, a statistical power analysis program, was used to determine the amount of individuals needed to recruit in the randomized controlled trial study. An effect size of 0.38, estimated by the abovementioned pilot study, informed us of the required sample size of 57 participants per intervention arm to achieve a power of 0.80. Descriptive baseline characteristics of groups are tabulated as means and SDs or as percentages. Differences between the four study arms in PA outcomes were tested according to the intention-to-treat approach [35]. Under this approach, study participants are analyzed as members of the treatment group to which they were randomized regardless of their adherence to the intended treatment. Data were inspected for normality. Participants were recruited throughout a period of one year and so the intervention took place in the four different seasons. To account for these differences, we calculated residuals of the PA variables to adjust our data for climatological variables such as mean daily temperature, mean daily precipitation and mean daylight hours. A residual is defined as the difference between the observed value of the PA variable and the predicted value (using sex, mean daily temperature, mean daily precipitation and mean daylight hours as predictors) [36]. In the results section, we will use the term ‘adjusted’ to refer to the PA residual. These PA residuals were analyzed using a two-way repeated measures ANCOVA with Group and Time as independent variables and each PA residual as dependent variable. All analyses took into account the baseline values of the PA outcome measures.

Our first hypothesis was that receiving feedback on the behavior would increase the activity level more after four weeks of intervention than receiving no feedback. To answer this first research question, we compared the PG against the MIG. Our second hypothesis was that receiving feedback on both the behavior and the behavioral outcome would be more effective after four week of intervention than receiving feedback on the behavioral outcome only. To answer this research question, we compared the PG against the DG. Our final hypothesis was that an intervention in which a need-supportive Personal Coach was used in combination with continuous self-monitoring would result in a stronger PA enhancement after four weeks of intervention compared with an intervention that only uses a technological device to provide measurement feedback. This last research question evaluated the added value of the Personal Coach and compared the CoachG with the DG. Analyses were performed using SAS 9.2 and significance level was set at *p* < 0.05.

## 3. Results and Discussion

### 3.1. Participants

All statistically significant differences between groups are shown: (a) tested using Chi-square; (b) tested using one-way ANOVA. Mean wear time of the armband was calculated for the total group of participants (e.g., 97.6% ± 4.3% at baseline, 68.6% ± 15.7% during the intervention and 67.8% ± 18.4% one week after the intervention). Wear time throughout and one week after the intervention significantly increased after imputing missing values for sleeping hours and for water activities. During the intervention, after data imputing, participants had an average SWA time of 96.4% ± 2.8% or data were available for an average of 1388 min or 23.1 h per day. One week after the intervention, the average SWA time was 93.5% ± 12.5%, which corresponds to 1346 min or 22.4 h per day. At post 1, participants already wore the armband for 5 weeks. Furthermore, at post 1, participants did not receive any form of feedback which could decrease their motivation to wear the armband. This could explain why wear time was significantly reduced at post 1 compared with baseline measurement.

Participants did not differ significantly at baseline between the four intervention arms with respect to sociodemographic, biological and behavioral characteristics (Table 1). Concerning the baseline PA outcome parameters measured by SWA, significant differences were observed for the mean steps/day and the mean PA level. Despite the random assignment of individuals to the four intervention arms, participants of the DG took significantly more steps/day (mean diff: 2239 ± 559 steps; *p* < 0.001) than the PG. The DG also had a significantly higher PA level (0.08 ± 0.03 METs; *p* < 0.05) compared with the PG which translates into a higher EE of 117 kcal per day for an individual with a body weight of 60 kg. No significant differences emerged for total daily EE, active EE and minutes of MVPA/day. Because of these differences between groups, all analyses took into account the baseline values of the PA outcome measures.

**Table 1:** Baseline participant characteristics.

Participants Characteristics	MIG		Pedometer G		Display G		Coaching G		*p*-Value
N (%)	54	(24.5)	55	(24.9)	56	(25.3)	56	(25.3)	
**SOCIODEMOGRAPHIC CHARACTERISTICS**									
Gender (%male) ^a^	46.3		45.5		46.4		44.6		0.997
Age in years (mean, SD) ^b^	41.2	(11.0)	43.3	(10.7)	44.3	(9.9)	40.7	(9.8)	0.211
Percent married (%) ^a^	41.5		64.2		60.4		46.3		0.529
Percent with children (%) ^a^	64.2		74.1		73.2		62.3		0.428
Percent white collar (%) ^a^	67.3		80.0		75.0		76.4		0.802
Percent higher education (%) ^a^	67.3		70.9		60.7		70.9		0.990
**BIOLOGICAL CHARACTERISTICS**									
Body Fat % (mean, SD) ^b^	28.4	(6.6)	29.4	(6.7)	29.6	(6.9)	29.4	(5.8)	0.753
BMI in kg/m² (mean, SD) ^b^	26.4	(3.3)	26.8	(4.2)	27.5	(3.9)	27.8	(4.5)	0.216
SBP in mmHg (mean, SD) ^b^	122.7	(16.0)	126.7	(17.4)	125.7	(16.4)	120.8	(15.0)	0.193
DBP in mmHg (mean, SD) ^b^	82.3	(10.3)	83.2	(11.4)	83.3	(10.4)	78.6	(8.5)	0.056
**BEHAVIORIAL CHARACTERISTIC**									
Smoking (% smokers) ^a^	15.1		7.4		7.1		11.1		0.557
Units alcohol/week (mean, SD) ^b^	1.60	(1.30)	1.60	(1.60)	1.60	(1.40)	1.70	(1.70)	0.978
FPACQ PA level (mean, SD) ^b^	1.68	(0.20)	1.63	(0.11)	1.71	(0.19)	1.68	(0.16)	0.148
**PA OUTCOME by SWA**									
Steps/day (mean, SD) ^b^	9855	(2983)	8840	(2306)	11079	(3431)	9978	(2940)	**0.001**
Min of MVPA/day (mean, SD) ^b^	116	(42)	101	(45)	118	(46)	107	(51)	0.186
Total daily EE (mean, SD) ^b^	2713	(402)	2634	(484)	2835	(533)	2751	(534)	0.187
Active daily EE (mean, SD) ^b^	595	(234)	516	(245)	638	(305)	578	(289)	0.125
PA level (mean, SD) ^b^	1.46	(0.14)	1.39	(0.16)	1.47	(0.16)	1.41	(0.17)	**0.030**

Notes: Values are means and standard deviations (SD) for continuous variables and percentages within intervention arms for categorical variables; Key: SD: standard deviation; MIG: Minimal Intervention Group; G: Group; BMI: Body Mass Index; SBP: systolic blood pressure; DBP: diastolic blood pressure; FPACQ: Flemish Physical Activity Computerized Questionnaire; MVPA: moderate to vigorous physical activity; PA level: Physical Activity Level; METs: metabolic equivalent of task; PA: Physical Activity; SWA: SenseWear Pro3 Armband; EE: Energy Expenditure.

**Figure 3:**
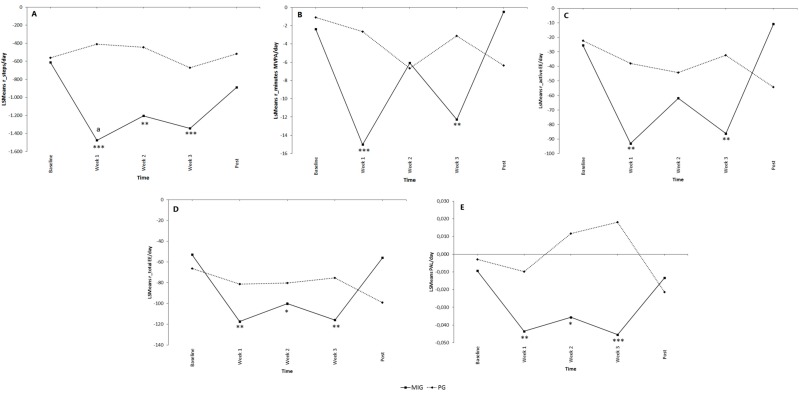
Residuals for steps (**A**); minutes of moderate to vigorous physical activity (**B**); total energy expenditure (**C**); active energy expenditure (**D**) and physical activity level (**E**) at baseline, week 1, week 2, week 3 and post 1 for the Minimal Intervention Group (MIG) and Pedometer Group (PG). Data are expressed as least square means (LSMeans). Between subjects and within subjects effects are shown. The letter a indicates a significant difference between MIG and PG at *p* < 0.05; an asterix (*) indicates a significant difference to baseline measurement within the MIG and PG; * *p* < 0.05; ** *p* < 0.01; *** *p* < 0.001.

### 3.2. Comparative Effectiveness of Feedback on Steps vs. No Feedback

The first research question referred to the comparative effectiveness of giving feedback by means of a pedometer (PG) *versus* not giving feedback (MIG). The letter a in Figure 3 indicates a significant difference between groups (PG *versus* MIG) for the different moments in time (week 1–3 and post). Our two-way ANCOVA analysis found no significant Group × Time interaction effect for the residuals of the different PA variables. A significant main effect for Group was found but only for the adjusted steps per day. More specifically, participants of the PG had a higher adjusted step count compared with the MIG. However, this difference in adjusted step count was only notable during the first week of the intervention (Mean diff: 1066 ± 345 steps; *p* = 0.033) while after the intervention, no significant difference between groups was shown. This indicates that no support could be found for Hypothesis 1.

The asterix in Figure 3 shows a significant difference compared with baseline measurement. The within subject test indicated that there was a significant Time effect within participants of the MIG for all adjusted PA parameters (*p* = 0.037). During the intervention, participants of the MIG showed a lower adjusted step count, total EE and PAL compared with baseline measurement. In week 1 and week 3, participants of the MIG also spent less adjusted time at MVPA and had a lower adjusted active EE compared with baseline measurement. No significant within-subjects effects were found across time for the PG.

### 3.3. Comparative Effectiveness of Feedback on Behavior (e.g., Steps) vs. Feedback on Both Behavior (e.g., Steps and Minutes of MVPA) and Behavioral Outcome (e.g., Total Calories Burned)

**Figure 4:**
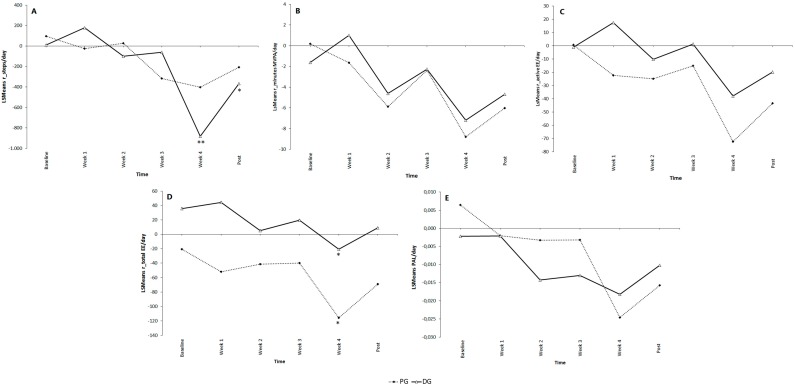
Residuals for steps (**A**); minutes of moderate to vigorous physical activity (**B**); total energy expenditure (**C**); active energy expenditure (**D**) and physical activity level (**E**) at baseline, week 1, week 2, week 3, week 4 and post 1 for the Pedometer Group (PG) and the Display Group (DG). Data are expressed as least square means (LSMeans). Within subject effects are shown. An asterix (*) indicates a significant difference to baseline measurement within the PG and DG; * *p* < 0.05; ** *p* < 0.01. Key: PA: Physical Activity; EE: Energy Expenditure.

Our second research question evaluated the effectiveness of measurement feedback given by a pedometer (PG) *versus* measurement feedback given by a SWA display (DG). Our two-way ANCOVA analysis found no significant Group × Time interaction effect for the residuals of the different PA variables (Figure 4). No significant main effect of Group was found. In other words, contrary to Hypothesis 2, no differences emerged between the PG and the DG for the residuals of the different PA parameters across time.

Our two-way ANCOVA revealed a significant within subject effect for adjusted steps per day and adjusted total EE. The asterix in Figure 4 indicates a significant difference with baseline measurement within the PG for the adjusted total EE and within the DG for the adjusted steps per day and the adjusted total EE. In contrast with our second hypothesis, compared with baseline, individuals of the PG and DG had a lower adjusted total EE the 4th week of the intervention (resp. mean diff: −95 ± 45 calories; *p* = 0.035 and mean diff: −57 ± 39 calories; *p* = 0.025). In addition, participants of the DG took fewer adjusted steps per day during the 4th week of the intervention (Mean diff: −893 ± 340 steps; *p* = 0.004) and one week after the intervention (Mean diff: −378 ± 342 steps; *p* = 0.033).

**Figure 5:**
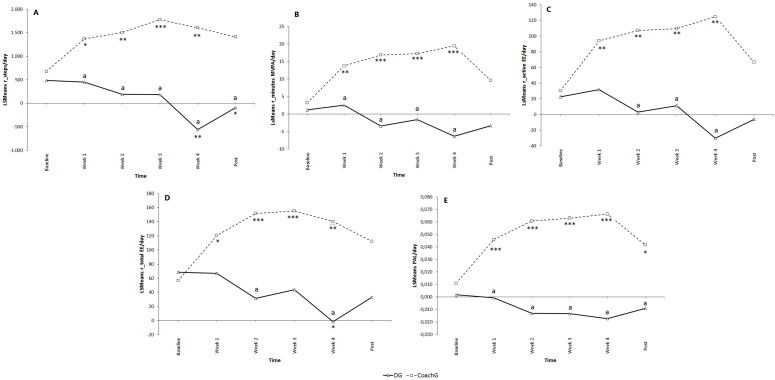
Residuals for steps (**A**), minutes of moderate to vigorous physical activity (**B**), total energy expenditure (**C**), active energy expenditure (**D**) and physical activity level (**E**) at baseline, week 1, week 2, week 3, week 4 and post 1 for the Display Group (DG) and the Coaching Group (CoachG). Data are expressed as least square means (LSMeans). Interaction effects between Group and Time are presented. The letter a indicates a significant difference between DG and CoachG at *p* < 0.05; an asterix (*) indicates a significant difference to baseline measurement within the DG and CoachG; * *p* < 0.05; ** *p* < 0.01; *** *p* < 0.001. Key: PA: Physical Activity; EE: Energy Expenditure.

### 3.4. Added Value of Personal Coaching

Finally, the third research question evaluated the added value of a Personal Coach. Because this research question wants to evaluate the surplus of coaching, we decided to compare PA parameters across time of individuals being supported by a coach (CoachG) in addition to the real-time feedback of the SWA display with participants only using the SWA display (DG). Figure 5 gives a graphical comparison of the two groups across time for the residuals of the different PA parameters.

Our two-way ANCOVA analysis found a significant Group × Time interaction effect for the residuals of the different PA variables. This means that both groups were changing over time but were changing in different ways. The letter a in Figure 5 indicates a significant difference between groups (CoachG and DG) for the different moments in time (week 1–4 and post). In line with Hypothesis 3, the adjusted step count, adjusted time spent at MVPA and adjusted PA level was higher during the intervention for individuals supported by a Personal Coach than for individuals who only used the SWA display. The CoachG also had a higher adjusted active EE and total EE the 2nd and 4th week of the intervention. One week after the intervention, participants of the Coach had a higher adjusted step count and adjusted PA level compared with the DG (resp. Mean diff: 1503 ± 324 steps; *p* = 0.002 and mean diff: 0.051 ± 0.016 METs; *p* = 0.026).

The asterix in Figure 5 shows a significant difference with baseline measurement within each group. Participants of the DG had a significantly lower adjusted step count and a lower adjusted total EE the 4th week of the intervention compared with their baseline values. Moreover, the DG had a lower adjusted step count one week after the intervention. Weekly being followed by a Personal Coach resulted in significant higher adjusted values for all PA parameters throughout the intervention compared with baseline measurement. One week after the intervention, when individuals no longer received any kind of feedback considering their steps, minutes spent at MVPA or total kcal, participants of the CoachG were still able to achieve a higher adjusted PA level (+0.031 ± 0.016 METs; *p* = 0.028).

## 4. Conclusions

The present study investigated the weekly changes in PA using different degrees of measurement feedback and the added value of need-supportive coaching on PA. The randomized controlled trial revealed a higher adjusted step count during the first week of the intervention for the PG compared with the MIG. This increase during the first week of the intervention indicates that receiving feedback on steps could prevent the decline in PA when receiving no feedback. Nevertheless, our first hypothesis was not supported given that one week after the intervention, no significant differences remained between the PG and the MIG.

The initial increase in daily steps after our respondents had started to use a pedometer is in line with previous research examining the issue of reactivity to self-monitoring devices. According to Matevey *et al.* [37], reactivity is “a change in behavior due to being monitored”. Clemes and colleagues [21] used covert monitoring (e.g., participants were unaware that their activity levels were being monitored) to investigate reactivity and reported that step counts increased in the group wearing the unsealed pedometers. However, the largest reactivity occurred in response to recording daily steps in a diary where step counts remained elevated for 1 week. In our controlled trial study, the difference in step count between the PG and the MIG was also only notable after the first week of the intervention. Furthermore, no significant differences in step count emerged across time within the PG. We concluded that if pedometers are to be used as an intervention tool, further strategies need to be used to sustain the motivational impact of pedometer use after the first week. Because of the rather high daily step count of our study sample at baseline (e.g., 8840 ± 2306 steps for PG), it is possible that there were ceiling effects in this measure. This could also explain why no significant increase in step count emerged.

Our second research question evaluated the effectiveness of measurement feedback on the behavior given by a pedometer (PG) *versus* measurement feedback on both the behavior and the behavioral outcome given by a SWA display (DG). No differences in the PA behavior between the two feedback modalities were found across time. In terms of self-monitoring, using a highly technological device with detailed feedback such as the SWA display seems of no greater value than a low-cost pedometer.

To our knowledge, no research in the PA domain has compared the effectiveness of setting goals on the behavior (e.g., steps and time spent at PA) *versus* setting goals on the behavioral outcome (e.g., total calories burned). This distinction between feedback on the behavior and feedback on the outcome appears to be common in behavioral weight loss programs. These programs typically use behavioral strategies such self-monitoring diet and exercise and self-weighing [38]. Consequently, feedback can be provided on the eating and activity behavior or on the outcome of how eating and exercise behavior can affect body weight.

Our randomized controlled trial showed a significant lower adjusted number of steps and adjusted total EE the 4th week of the intervention for the DG and a significant lower adjusted total EE for the PG compared with baseline measurement. This is in line with a study of Godino [39] who investigated whether or not feedback on PA stimulated behavior change. These researchers found that the provision of personalized feedback about PA was not associated with changes in PA after eight weeks. They concluded that although feedback may moderately increase awareness of behavior, it is not sufficient to change behavior in the short-term but might enhance the effects of a more intensive behavior change intervention.

Our third and final research question investigated the added value of a need-supportive and one-to-one coaching. As predicted, the results demonstrated that having a weekly meeting with a coach leads to a higher and sustained increase in PA compared with using only a self-monitoring device (e.g., SWA display). At the end of the intervention, large differences were observed in favor of the CoachG for the adjusted step count and the adjusted PA level. Furthermore, individuals receiving need-supportive coaching were able to increase their adjusted steps per day, adjusted time spent at MVPA, adjusted total and active EE and adjusted PA level throughout the intervention and showed a higher adjusted PA level one week after the intervention.

Previous research evaluated the effectiveness of need-supportive coaching on self-reported activity levels. For example, Van Hoecke *et al.* [40] designed a need-supportive coaching program for university employees, consisting of five individual contact moments (*i.e.*, an intake session, three follow-up contacts and an out-take session). Their study showed a significant increase in self-reported mild, moderate, vigorous and total PA over a period of four months. However, other studies that compared different degrees of intensities in coaching suggested a possible benefit for the most intensive interventions compared with a briefer intervention [41]. In our study, the coaching consisted of four weekly 30-min sessions over a 4-week intervention period. It would be interesting to study if even a lower intensity of coaching could provide the same results or if, as suggested by Opdenacker *et al.* [42], brief telephonic prompting is as effective as individual counseling.

To our knowledge, only a few studies have been conducted in the past that explore the effect of need-supportive coaching on PA levels when PA was measured using an objective self-monitoring device [43]. In the present study, coaching was provided in combination with using a high technological SWA display. However, the current study design did not allow investigating if coaching without using the display has the same beneficial effects on PA behavioral change. Our results showed that using the display without coaching did not alter the PA behavior during the intervention. Nevertheless, the usefulness of the SWA display should not be underestimated. In the one-to-one conversations with the Coach, data from the SWA was exported and used to objectively and graphically present the PA behavior of the participants. It is possible that the graphical display of information independently contributed to the increase in PA. Furthermore, the SWA display served as a tool to personalize the exercise targets and to provide motivational messages when the individualized targets were met. It would be interesting to investigate whether using a low-cost pedometer as feedback instrument in combination with a coach or only having a weekly follow-up with a coach without using a monitoring device would increase the PA level of participants. Furthermore, given the previous points raised, future studies could investigate using video-based coaching sessions instead of real-life coaching sessions would have the same effect. More specifically, participants could wear the SWA for one week and consequently transfer the physical activity data on their own PC. Participants can graphically see their physical activity pattern (energy expenditure, steps, minutes of MVPA), select events or receive an evaluation of their physical activity level. When computer-tailored advice is delivered prior to the online coaching session, it can reduce the time required from a coach to provide feedback, therefore minimizing the time and financial cost to conduct the intervention. The addition, the online coaching sessions can offer the possibility to personalize and interpret the results of the physical activity assessment. From the perspective of the Self-Determination Theory, it is possible that an individual would feel less related with a web-based coach compared with a real-life coach. However, the advances in internet technology and broadband capacity can allow the coaching sessions to be delivered via free online video-calling programs (e.g., Skype), which enable the participant to view the coach whilst engaging in a verbal discussion resulting in a higher level of relatedness.

### 4.1. Limitations and Strengths

This study has several strengths. First, we recruited a relatively large population-based sample with a very high participant retention, which did not differ between intervention arms (97%). Second, we took into account climatological data such as daily temperature, daily precipitation and daylight hours to calculate the PA residuals. To our knowledge, this is the first study using these climatological data. Third, a valid objective measure of PA was used in this trial. Fourth, mean wear time of the SWA was high at baseline, during the intervention and in the week after the intervention. Fifth, this study is unique because it quantifies in detail the weekly PA behavioral change measured during the intervention period instead of only pre-post PA outcome measures. Finally, to our knowledge, this is the first study that compared the effectiveness of feedback on the behavior and feedback on both the behavior as the behavioral outcome against a no feedback condition and at the same time evaluated the added value of need-supportive coaching.

Despite the abovementioned strengths, four limitations of the study should also be considered. First, to allow a direct comparison between the different types of real-time feedback, we chose not to have a ‘pure’ control arm throughout the study. We believed that it would not be ethical and practical to ask someone who had volunteered for an intervention study and who had indicated a wish to change his/her activity behavior to remain on a waiting list control condition for 12 months. However, we did use a Minimal Intervention Group in which only one contact moment with a coach was allowed immediately after baseline measurement. This group did not receive any feedback during the intervention. We recognize however that the lack of a ‘pure’ control condition throughout constitutes a limitation of the study.

A second limitation is the difference in PA level of the study groups at baseline. The participants that had been randomized to the DG were slightly more active at baseline than those randomized to the PG. By using the baseline measurement as covariate in our analyses, we took into account the effect of this baseline difference between group comparisons.

A third limitation is the high baseline activity level of the sample, at least with respect to the daily number of steps measured by the SWA. The ceiling effect may be more apparent for the DG compared with the PG which could make these participants less aware of the need to change their number of steps given the feedback they received regarding their seemingly high step count. On the other hand, it can be questioned whether the SWA is accurate in counting steps. In our opinion, no study has ever investigated the validity of SWA in estimating steps during free-living activities. Laboratory studies that investigated the accuracy of SWA during walking in healthy adults showed that the SWA was reasonably accurate in counting steps [44,45]. Considering that the participants of the PG simultaneously wore a Digiwalker and the SWA during the 4-week intervention period, the present study offered the possibility to validate the SWA in counting steps during free-living activities. Additional analyses suggest that SWA overestimates compared with the daily step count of the Digiwalker (10,237 ± 2802 *vs.* 8856 ± 2811, mean diff: 1382 ± 1328 steps; *p* < 0.0001). This potential overestimation of SWA advocates that the baseline activity level of our study sample was not that high, resulting in approaching the target population of inactive working adults.

A final limitation is the underestimation of SWA at higher intensities. Previous research showed an error in the estimation of the energy expenditure above intensities of 10 METs [46]. However, it should be noted that the contribution of high intensity exercise to the total daily activity level is negligible under normal daily living conditions [47]. Consequently, the SWA can be used to determine the daily physical activity level of an individual.

### 4.2. Conclusions

In summary, different behavioral strategies such as self-monitoring, goal setting and providing feedback have been shown to be effective in promoting an active lifestyle. However, little is known about the effectiveness of different degrees of feedback and the added value of coaching on the enhancement of PA. This study is the first to examine the efficacy of different degrees of feedback in promoting PA behavioral change. The difference in the adjusted step count between using a pedometer and receiving no feedback was only notable at the start of the intervention. Our findings suggest that by using a simple self-monitoring device, no significant increase in PA behavior occurred. It appears that adding coaching successfully increases steps, minutes of MVPA, active EE and the PA level not only during but also the week after a 4-week intervention period. This study provides an important contribution to the area of public health as it provides evidence that minimal contact interventions (through the form of a SWA display and four contact meetings with a coach) have the capacity to produce behavior change. It should be noticed that these results represent acute responses to the intervention and do not constitute long-term behavioral change. Follow-up data from the trail will ultimately provide useful insights.

## References

[1] Powell K.E., Paluch A.E., Blair S.N. (2011). Physical activity for health, what kind? How much? How intense? On top of what?. Annu. Rev. Public Health.

[2] Vuori I.M., Oja P., Paronen O. (1994). Physically active commuting to work—Testing its potential for exercise promotion. Med. Sci. Sports Exerc..

[3] Andersen R.E., Blair S.N., Cheskin L.J., Bartlett S.J. (1997). Encouraging patients to become more physically active, the physician’s role. Ann. Intern. Med..

[4] Dunn A.L., Marcus B.H., Kampert J.B., Garcia M.E., Kohl H.W., Blair S.N. (1999). Comparison of lifestyle and structured interventions to increase physical activity and cardiorespiratory fitness, a randomized trial. JAMA.

[5] Warburton D.E., Nicol C.W., Bredin S.S. (2006). Health benefits of physical activity, the evidence. CMAJ.

[6] Giannuzzi P., Mezzani A., Saner H., Björnstad H., Fioretti P., Mendes M., Cohen-Solal A., Dugmore L., Hambrecht R., Hellemans I. (2003). Physical activity for primary and secondary prevention. Position paper of the Working Group on Cardiac Rehabilitation and Exercise Physiology of the European Society of Cardiology. Eur. J. Cardiovasc. Prev. Rehabil..

[7] Caspersen C.J., Powell K.E., Christenson G.M. (1985). Physical activity, exercise, and physical fitness, definitions and distinctions for health-related research. Public Health Rep..

[8] Wang J., Sereika S.M., Chasens E.R., Ewing L.J., Matthews J.T., Burke L.E. (2012). Effect of adherence to self-monitoring of diet and physical activity on weight loss in a technology-supported behavioral intervention. Patient Prefer. Adherence.

[9] Lippke S., Ziegelmann J.P. (2008). Theory-based health behavior change: Developing, testing, and applying theories for evidence-based interventions. Appl. Psychol. Int. Rev..

[10] De Silva D. (2011). Evidence, Helping People Help Themselves.

[11] Avery L., Flynn D., van Wersch A., Sniehotta F.F., Trenell M.I. (2012). Changing physical activity behavior in type 2 diabetes, a systematic review and meta-analysis of behavioral interventions. Diabetes Care.

[12] Heath G.W., Parra D.C., Sarmiento O.L., Andersen L.B., Owen N., Goenka S., Montes F., Brownson R.C., Lancet Physical Activity Series Working Group (2012). Evidence-based intervention in physical activity, lessons from around the world. Lancet.

[13] Conroy M.B., Yang K., Elci O.U., Gabriel K.P., Styn M.A., Wang J., Kriska A.M., Sereika S.M., Burke L.E. (2011). Physical activity self-monitoring and weight loss: 6-Month results of the SMART trial. Med. Sci. Sports Exerc..

[14] Michie S., Ashford S., Sniehotta F.F., Dombrowski S.U., Bishop A., French D.P. (2011). A refined taxonomy of behaviour change techniques to help people change their physical activity and healthy eating behaviours, the CALO-RE taxonomy. Psychol. Health.

[15] Bassett D.R., Dinesh J. (2010). Use of pedometers and accelerometers in clinical populations, validity and reliability issues. Phys. Ther. Rev..

[16] Tudor-Locke C., Craig C.L., Brown W.J., Clemes S.A., de Cocker K., Giles-Corti B., Hatano Y., Inoue S., Matsudo S.M., Mutrie N. (2011). How many steps/day are enough? For adults. Int. J. Behav. Nutr. Phys. Act..

[17] Kang M., Marshall S.J., Barreira T.V., Lee J.O. (2009). Effect of pedometer-based physical activity interventions, a meta-analysis. Res. Q. Exerc. Sport.

[18] Bravata D.M., Smith-Spangler C., Sundaram V., Gienger A.L., Lin N., Lewis R., Stave C.D., Olkin I., Sirard J.R. (2007). Using pedometers to increase physical activity and improve health: A systematic review. JAMA.

[19] Baker G., Mutrie N., Lowry R. (2011). A comparison of goals set in steps using a pedometer and goals set in minutes, A randomized controlled trial. Int. J. Health Promot. Educ..

[20] Clemes S.A., Deans N.K. (2012). Presence and duration of reactivity to pedometers in adults. Med. Sci. Sports Exerc..

[21] Rennie K.L., Wareham N.J. (1998). The validation of physical activity instruments for measuring energy expenditure, problems and pitfalls. Public Health Nutr..

[22] Wilson D., Haglund B. Worklplace performance monitoring: Analysing the combination of physiological and environmental sensory inputs. Proceedings of the IEE Eurowearable.

[23] Jones V., Bults R., de Wijk R., Widya I., Batista R., Hermens H. (2011). Experience with using the sensewear BMS sensor system in the context of a health and wellbeing application. Int. J. Telemed. Appl..

[24] Deci E.L., Eghrari H., Patrick B.C., Leone D.R. (1994). Facilitating internalization: The self-determination theory perspective. J. Personal..

[25] Ryan R.M., Deci E.L. (2000). Self-determination theory and the facilitation of intrinsic motivation, social development, and well-being. Am. Psychol..

[26] Van Hoye K., Boen F., Lefevre J. (2012). The effects of physical activity feedback on behavior and awareness in employees, study protocol for a randomized controlled trial. Int. J. Telemed. Appl..

[27] Saris W.H., Blair S.N., van Baak M.A., Eaton S.B., Davies P.S., di Pietro L., Fogelholm M., Rissanen A., Schoeller D., Swinburn B. (2003). How much physical activity is enough to prevent unhealthy weight gain? Outcome of the IASO 1st Stock Conference and consensus statement. Obes. Rev..

[28] FAO/WHO/UNO (1985). Energy and Protein Requirements.

[29] World Health Organization (2010). Global Recommendations on Physical Activity for Health.

[30] Jakicic J.M., Marcus M., Gallagher K.I., Randall C., Thomas E., Goss F.L., Robertson R.J. (2004). Evaluation of the SenseWear Pro Armband to assess energy expenditure during exercise. Med. Sci. Sports Exerc..

[31] St-Onge M., Mignault D., Allison D.B., Rabasa-Lhoret R. (2007). Evaluation of a portable device to measure daily energy expenditure in free-living adults. Am. J. Clin. Nutr..

[32] Ainsworth B.E., Haskell W.L., Whitt M.C., Irwin M.L., Swartz A.M., Strath S.J., O’Brien W.L., Bassett D.R., Schmitz K.H., Emplaincourt P.O. (2000). Compendium of physical activities, an update of activity codes and MET intensities. Med. Sci. Sports Exerc..

[33] Schneider P.L., Crouter S.E., Bassett D.R. (2004). Pedometer measures of free-living physical activity, comparison of 13 models. Med. Sci. Sports Exerc..

[34] Crouter S.E., Schneider P.L., Karabulut M., Bassett D.R. (2003). Validity of 10 electronic pedometers for measuring steps, distance, and energy cost. Med. Sci. Sports Exerc..

[35] Fisher L.D., Dixon D.O., Herson J., Frankowski R.K., Hearron M.S., Peace K.E.F. (1990). Intention to treat in clinical trials. Statistical Issues in Drug Research and Development.

[36] Cook D., Weisberg S. (1982). Residuals and Influence in Regression.

[37] Matevey C., Rogers G.R., Dawson E., Tudor-Locke C. (2009). Lack of reactivity during pedometer self-monitoring in adults. Meas. Phys. Educ. Exerc. Sci..

[38] Burke L.E., Wang J., Sevick M.A. (2011). Self-monitoring in weight loss, a systematic review of the literature. J. Am. Diet. Assoc..

[39] Godino J.G., Watkinson C., Corder K., Marteau T.M., Sutton S., Sharp S.J., Griffin S.J., van Sluijs E.M.F. (2013). Impact of personalised feedback about physical activity on change in objectively measured physical activity (the FAB study): A randomised controlled trial. PLoS ONE.

[40] Van Hoecke A.S., Delecluse C., Opdenacker J., Lipkens L., Martien S., Boen F. (2013). Long-Term effectiveness and mediators of a need-supportive physical activity coaching among Flemish sedentary employees. Health Promot. Int..

[41] Ogilvie D., Foster C.E., Rothnie H., Cavill N., Hamilton V., Fitzsimons C.F., Mutrie N. (2007). Interventions to promote walking: Systematic review. BMJ.

[42] Opdenacker J., Boen F. (2008). Effectiveness of face-to-face *versus* telephone support in increasing physical activity and mental health among university employees. J. Phys. Act. Health.

[43] Sebire S.J., Standage M., Vansteenkiste M. (2011). Predicting objectively assessed physical activity from the content and regulation of exercise goals, evidence for a mediational model. J. Sport Exerc. Psychol..

[44] Dwyer T.J., Alison J.A., McKeough Z.J., Elkins M.R., Bye P.T. (2009). Evaluation of the SenseWear activity monitor during exercise in cystic fibrosis and in health. Respir. Med..

[45] Hill K., Dolmage T.E., Woon L., Goldstein R., Brooks D. (2010). Measurement properties of the SenseWear armband in adults with chronic obstructive pulmonary disease. Thorax.

[46] Van Hoye K., Mortelmans P., Lefevre J. (2014). Validation of the SenseWear Pro3 Armband using an incremental exercise test. J. Strenght Cond. Res..

[47] Scheers T., Philippaerts R., Lefevre J. (2012). Patterns of physcial activitya and sedentary behavior in normal-weight, overweight and obese adults, as measured with a portable armband device and an electornic diary. Clin. Nutr..

